# Channel-forming discharge based on the extreme value identification of sediment-carrying capacity index

**DOI:** 10.1038/s41598-024-56528-x

**Published:** 2024-03-09

**Authors:** Hua Ge, Lingling Zhu

**Affiliations:** 1 Changjiang River Scientific Research Institute, Wuhan, 430010 China; 2 Key Laboratory of River and Lake Regulation and Flood Protection in the Middle and Lower Reaches of Changjiang River, Ministry of Water Resources, Wuhan, 430010 China; 3 Bureau of Hydrology, Changjiang Water Resources Commission, Wuhan, 430010 China

**Keywords:** channel-forming discharge, Suspended sediment-carrying capacity, Three Gorges Reservoir, Flood regulation of reservoirs, The middle and lower reaches of the Yangtze River, Climate sciences, Hydrology

## Abstract

Channel-forming discharge (*D*_cf_) is an important parameter in river management and reservoir flood regulation. Applying the methods for calculating *D*_cf_ to reaches downstream reservoirs characterized by drastic changes in water and sediment conditions and long-term scouring status is difficult. Based on the riverbed-shaping principle of sediment-laden water flow, while simultaneously considering the active action of water flow and response of the riverbed, this study proposes a new method for calculating *D*_cf_ by identifying the extreme value of the suspended sediment-carrying capacity index. The application of this method to the middle and lower reaches of the Yangtze River showed that after the impoundment of the Three Gorges Reservoir, *D*_cf_ in this section was reduced by an amplitude between 2500 and 4700 m^3^/s. The results can be used to guide the operation of the Three Gorges Reservoir and the management of the middle and lower reaches of the Yangtze River, thus providing reference for other river channels downstream of the reservoir.

## Introduction

The riverbed shaping effect of the channel-forming discharge (*D*_cf_) is equivalent to a multi-year flow discharge (*D*_f_) process. It is closely related to flood magnitude, process, duration, and suspended sediment concentration (SSC) and its combination with the water flow process and river boundary conditions. It is not only an important parameter that reflects the geometry of the river^1^, but also an indicator of the SSC of the river channel. It is the foundation of river evolution analysis and plays an important role in river planning and regulation^2,3^.

The *D*_f_ process when the water level (*Z*_f_) is flush with the surface of the sandbar in the river reach is widely applied in the determination of *D*_cf_ in plain rivers^4–6^, and is referred to as the bankfull (BK) method. Certain viewpoints suggest that the peak *D*_f_ at a certain frequency has a greater effect on riverbed evolution^7^; therefore, it can be used as *D*_cf_^8^. This method is called the guaranteed rate (GR) method. The improvement in this method mainly focuses on the optimization of the guarantee rate^9^. Because the adjustment of the riverbed ultimately depends on incoming water and sediment, these two factors have been adopted to analyze *D*_cf_ in numerous studies. For example, the effective discharge concept has been used to characterize the Cumberland Basin streams^10^, Liaohe River^11^, and middle Yangtze River^12,13^. Makayev adopted discharge as the *D*_cf_ when the product of the suspended sediment-carrying capacity (SSCC) and its corresponding *D*_f_ guarantee rate was maximized^14,15^, which is referred to as the MK method in this study. The results of the MK method were basically the same as those of the effective sediment transport discharges in Heilongjiang River^16^, but closer to the results of the BK method in the Huai River^17^ and Han River^18^. In the Yellow River, the difference between the MK and BK methods was relatively obvious^19^. In the fluctuating backwater area of Three Gorges Reservoir (TGR), the MK method results were similar to those of the GR method^20^. In addition, the method based on the curve of the product of the SSC and its frequency was proposed. This method is simpler and its results for the Yellow River were not substantially different from those of the MK method^21^. The multiyear average value of the daily averaged flood *D*_f_ was also used as *D*_cf_; however, it is not suitable for urban river channels^22^.

The *D*_cf_ values calculated using the methods above differed from each other^23,24^, and have different adaptabilities to different rivers^25,26^. The BK method is more suitable for channels with relatively stable sandbars and deep pools, whereas the MK method generally yields two *D*_cf_ values, which do not reflect drastic changes in water and sediment conditions^27^. Optimized methods have also been developed. In the Yellow River, *D*_cf_ is defined as the first and second *D*_cf_^28,29^ and has been used to guide the regulation of the Xiaolangdi Reservoir^30^. The introduction of parameters, such as SSCC^31^, SSC frequency^27,32^, riverbed balance^33^, sliding analysis correction^34^, and the ratio of squared SSC to *D*_f_^35^ has also optimized the calculation of *D*_cf_. In addition, some methods are based on the optimization of certain parameters, such as the use of a formula for calculating the bed load sediment transport rate^18^, the selection of representative periods, and the index in the MK method^36^. Certain methods are based on the distribution characteristics of flow frequency curves^11^, numerical simulation, and deep learning models^37^.

In summary, the above methods have mainly been applied to rivers with a high SSC^38–40^. For those downstream of reservoirs with low SSC, the adjustment of the medium flood frequency and the relationship between water and sediment after reservoir regulation will have a significant impact on *D*_cf_^41,42^. Numerical experiments have also shown that sediment transport patterns, channel morphology, *D*_f_ variation, and recording length interact with each other, thereby affecting the estimation of *D*_cf_^43^ and resulting in obvious changes in *D*_cf_^44^. The application of existing methods to reaches downstream of reservoirs characterized by drastic changes in water and sediment conditions and long-term scouring status is difficult and necessitates improvement. Based on the fundamentals of riverbed deformation and the background of channel reclamation downstream of reservoirs, this study proposes a method for accurately identifying the *D*_cf_ in view of the SSCC index in those altered flow and sediment regime conditions, thus enabling the scientific scheduling of reservoirs and ensuring the shaping effect of water flow downstream of the reservoirs, such as in the TGR.

## Materials and methods

### Study area

With the commissioning of the TGR and a series of cascade reservoirs in the upper Yangtze River, hydrological elements, such as the D_f_ magnitude, temporal and spatial distribution, and the SSC entering the middle and lower reaches of the Yangtze River (MLYR) have changed, resulting in significant changes in the evolution of the riverbed downstream of the reservoirs^45^. The first and foremost change was a reduction in the flood peak. For example, in 2010 and 2012, the peak floods flowing into the TGR reached 70,000 m^3^/s and 71,200 m^3^/s, respectively, but the *D*_f_ released by the reservoir was controlled to within 45,000 m^3^/s. The second was the increase in the medium water duration. From 2008 to 2012, the duration of *D*_f_ between 25,000 and 40,000 m^3^/s in July and August flowing into the MLYR, represented by the Yichang hydrographic station extended from 121 to 144 days. In addition, the increased *D*_f_ during the dry seasons before flood season and decreased *D*_f_ after flood season constitute changes in the temporal and spatial distribution of water flow.

The change in the sediment is relatively obvious, and was manifested by a significant reduction in the SSC and sediment particle size^46^. The average annual transported sediment amounts at the Yichang, Hankou, and Datong hydrographic stations from 2003 to 2016 were 0.381 × 10^8^ t, 1.03 × 10^8^ t, and 1.4 × 10^8^ t, respectively, which were 92%, 74%, and 67% less than those before storage in the TGR. The relationship between water and sediment was also altered, as shown by a lower SSC under the same *D*_f_. In future, following the completion and utilization of reservoirs in the upper reaches of the Yangtze River, the amount of sediment entering the MLYR will be further reduced, and the riverbed, not only the reach in the middle Yangtze River^47^, but even the estuarine areas^48,49^ will undergo a longer period of scouring adjustment.

**Figure 1:**
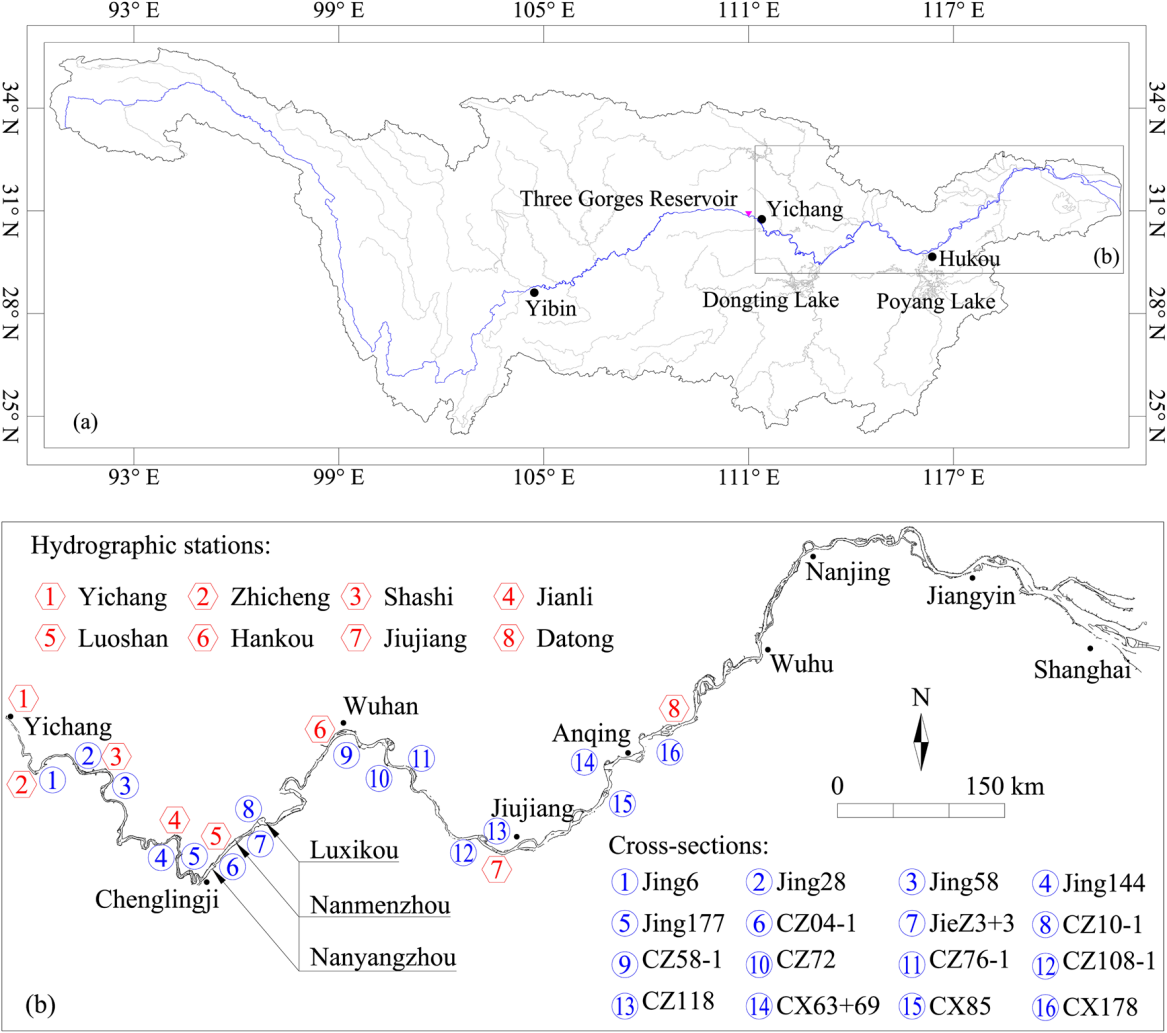
Study area: (**a**) the Yangtze River Basin; (**b**) the middle and lower reaches of the Yangtze River. This figure was modified from the picture in reference^50^, which includes the annotation of geographical information, such as boundary points Yibin, Yichang and Hukou among the upper, middle, and lower reaches of the Yangtze River, as well as the other important geographical locations, the hydrographic stations and cross-sections.

Based on the information above, the MLYR (Fig. 1) is a typical river section affected by reservoir impoundment. Therefore, it was selected as the study area and will provide reference for other river reaches in downstream reservoirs.

### Data

The data used in this study mainly consisted of cross-sectional terrain data measured at different sections of the MLYR. This collection included topographic data from 16 sections measured during the dry seasons since 1981. To calculate the hydraulic elements, such as the flow velocity (*U*) and water depth (*H*) of the sections, daily averaged *D*_f_, SSC, and *Z*_f_ data from the eight hydrographic stations upstream and downstream of the sections were collected simultaneously. In addition, certain river sections, such as the Nanyangzhou, Nanmenzhou, and Luxikou reaches, were selected to analyze the relationship between the hydraulic elements. These typical river sections have not yet implemented large-scale sandbar protection projects, and their evolution is almost natural, which is likely to reflect the shaping effect of water and sediment conditions on the riverbed. The positions of the cross sections and hydrographic stations are shown in Fig. 1b. All data were sourced from the Hydrological Bureau of the Yangtze River Water Resources Commission, and detailed information is provided in Table 1.

**Table 1:** Data information.

Data type	Measurement	Source
Daily averaged *D*_f_, SSC and *Z*_f_ of the 8 hydrographic stations	1981–2022	Hydrological Bureau of the Yangtze River Water Resources Commission
Topographic data of the 16 cross-sections	Since 1981	

### Method

#### Physical background of riverbed deformation

Riverbed deformation is the result of the sediment-laden water flow movement, which can be expressed in the following formula^51^:

$$\rho_{s} \frac{{\partial z_{b} }}{\partial t} = \alpha \omega \left( {S - S^{*} } \right)$$

where $\rho_{s}$ is the dry weight of the sediment, $z_{b}$ is the riverbed deformation amplitude over time $t$, $\alpha$ is the saturation recovery coefficient, $\omega$ is the sediment particle settling velocity, $S$ is the SSC, and $S^{*}$ is SSCC of the water flow, which is determined by the index $U^{3} /H$. Among them, $\rho_{s}$ and $\omega$ are the basic properties of sediment, and $\alpha$ has a certain range of values, which can be determined by the calibration of measured data. Therefore, riverbed deformation mainly depends on the contrast between $S$ and $S^{*}$. When $S$ is greater than $S^{*}$, the riverbed is silted, and vice versa. When the two were comparable, the riverbed was in equilibrium. This indicates that the *D*_f_ corresponding to the maximum SSCC has the most significant bed shaping effect.

#### Method to recognize ***D***_cf_ based on the SSCC index

Scouring under long-term subsaturated flow is the current and likely the considerable future state of the riverbed downstream of reservoirs. Channel development is the result of the combined effect of water flow, sediment, and channel boundaries, and the calculation of *D*_cf_ should consider these two factors simultaneously. There have been attempts to do so in the past, but when applied to rivers downstream of reservoirs, certain factors may change from primary to secondary, such as SSC. In rivers downstream of the reservoir, particularly downstream of the cascade reservoirs, the SSC is generally extremely small. For example, the annual sediment amount delivered into the MLYR has been below 1 × 10^7^ t since 2014^46^, and the average value from 2014 to 2016 has been reduced by more than 98% compared to the multi-year average value before storage in the TGR. The incoming SSC was significantly mismatched with the SSCC. The shaping of the river channel by water flow is more dependent on its capacity to pick up sediment from the riverbed.

**Figure 2:**
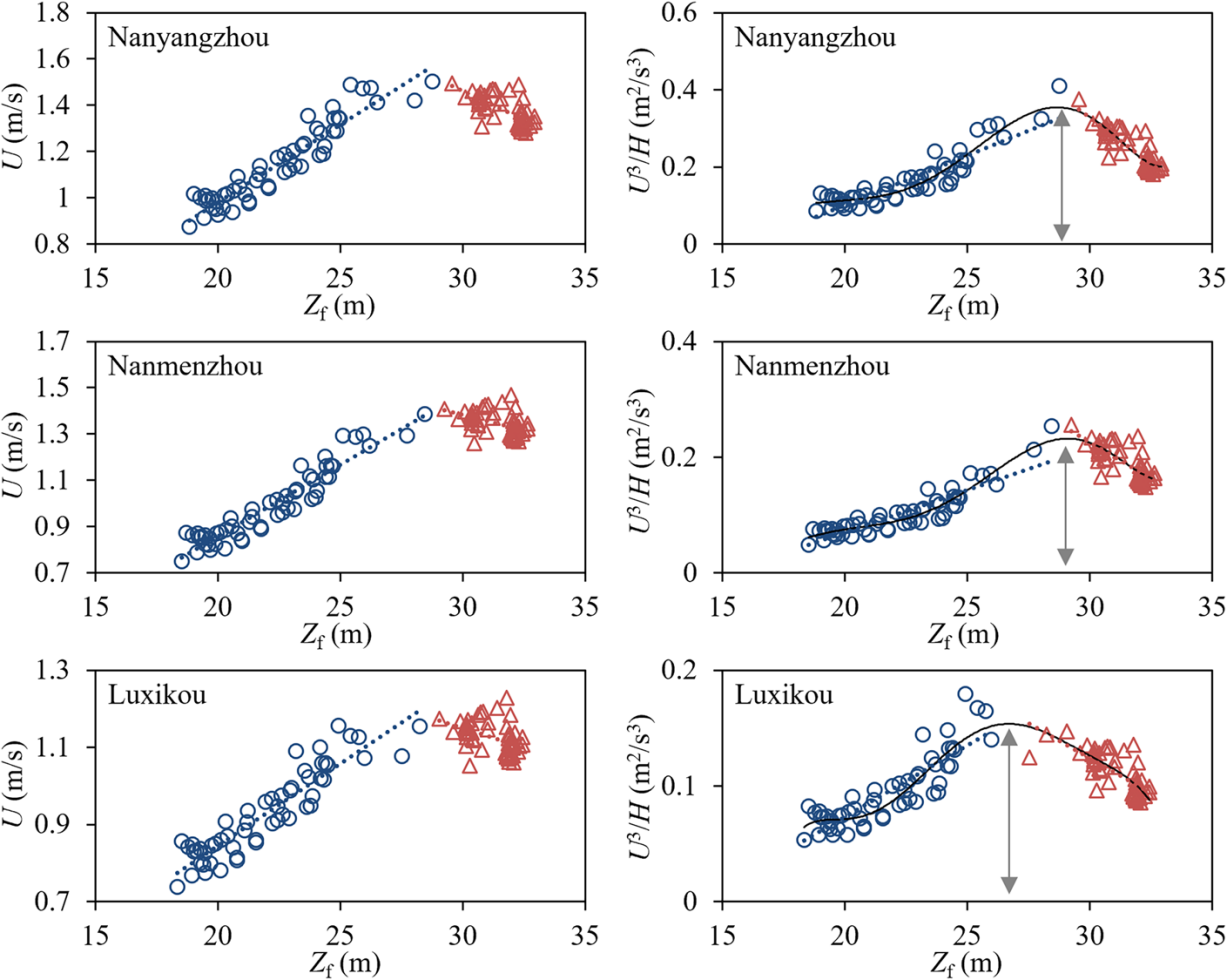
Relationship between *Z*_f_, *U*, and SSCC index in typical river sections.

However, the SSCC does not always increase with the *D*_f_ but starts to decrease upon exceeding a certain value. Figure 2 shows the correlation between the *Z*_f_, $U$, and the SSCC index $U^{3} /H$ in several typical sections of the MLYR that have high sandbars and are minimally affected by human activities. As the *Z*_f_ increases, $U$ does not continuously increase, but turns at a particular *Z*_f_. This turning point is also the extreme value point of $U^{3} /H$, which indicates that the ability of the water flow to deliver sediment and shape the riverbed reaches its maximum value. The *D*_f_ corresponding to the *Z*_f_ at this extreme point can be considered to be *D*_cf_. The specific steps of this method are as follows.

**Figure 3:**
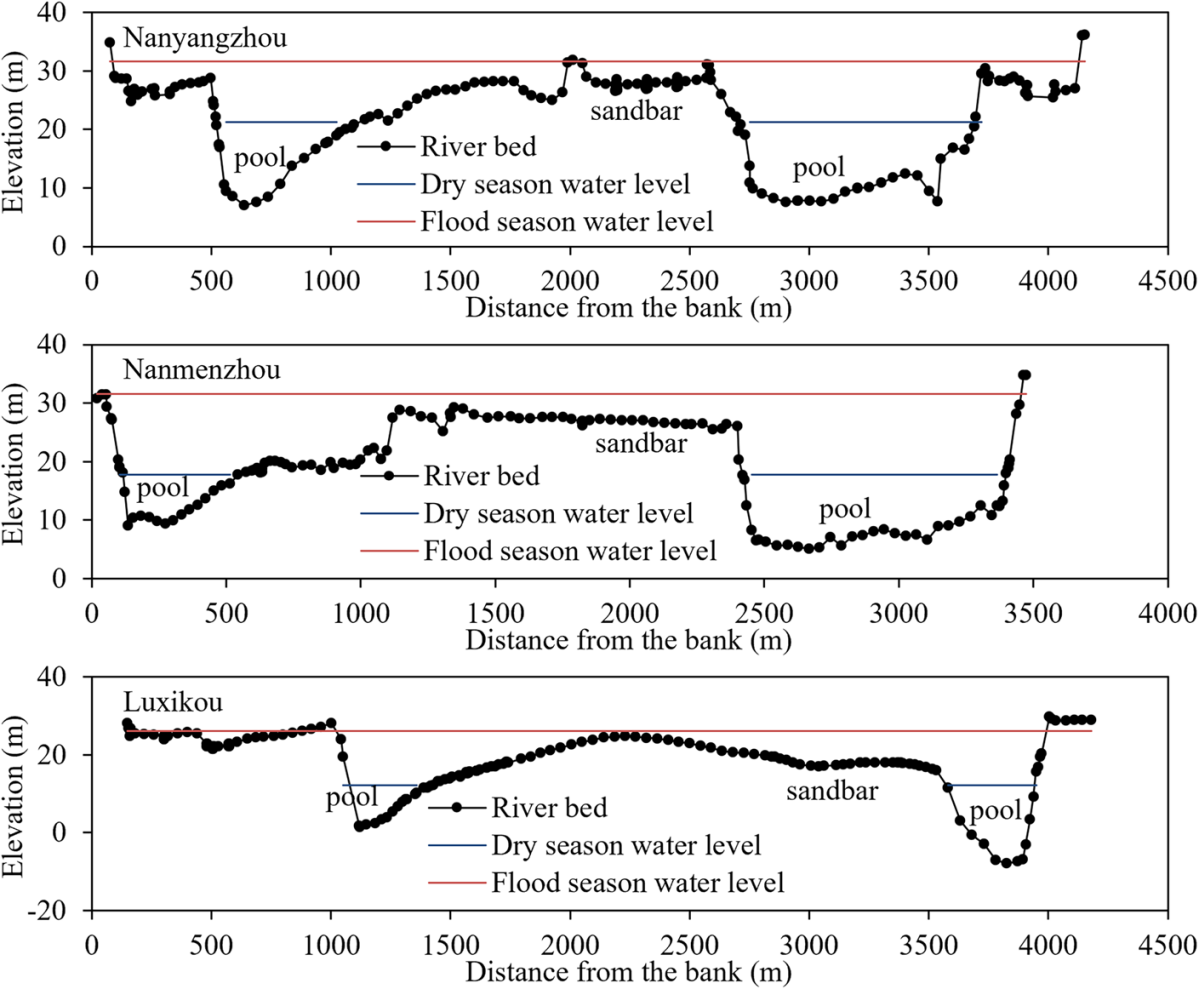
Cross-section morphology of selected typical river sections in this study.

First, we selected cross sections with clear sandbars and deep pools that were less affected by human activities within the river channel and collected their terrain observation data. The selected river sections need to have a clear binary structure of sandbars and deep pools. Figure 3 shows the cross-sectional morphology of several typical river sections selected in this study. Evidently, the cross-section distribution of these river sections has obvious sandbars and deep pools. During the dry season, water flows in deep pools, while during the flood season, water flows over the sandbars and fills the cross-section. The relatively little disturbance caused by human activities refers to the lack of bank and sandbar protection. In this case, the river bed evolution is mainly influenced by water, sediment, and riverbed boundary conditions.

**Figure 4:**
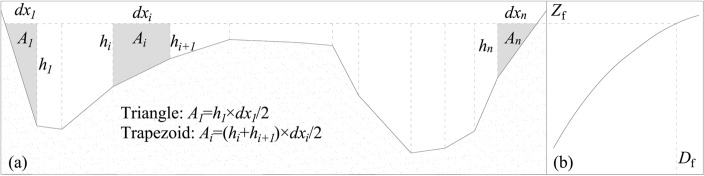
Diagram of hydraulic element calculation for cross-section: (**a**) cross-sectional terrain; (**b**) *Z*_f_ ~ *D*_f_ relationship curve.

Second, we calculated $U$ and $U^{3} /H$ of the cross sections at different *Z*_f_. For a given cross-section, it can be divided into multiple trapezoidal or triangular elements based on the measured points as shown in Fig. 4a. When *Z*_f_ is determined, the area $\left( {A_{i} } \right)$ of each sub section is calculated using the triangle or trapezoidal area formula. The total area ($A$) and width ($B$) of the section corresponding to *Z*_f_ can be obtained by accumulating $A_{i}$ and $dx_{i}$ of the sub sections. According to the *Z*_f_ ~ *D*_f_ relationship curve as shown in Fig. 4b, the *D*_f_ corresponding to *Z*_f_ can be found, then $U$ and $H$ can be calculated by $U = D_{f} /A$ and $H = A/B$. The correlation diagram between $U$, $U^{3} /H,$ and *Z*_f_ is shown in Fig. 2.

Third, the *Z*_f_ corresponding to the extreme value points of $U$ and $U^{3} /H$ on the correlation diagram were determined. Determining this extremum necessitates the use of polynomials to fit the scatter points as shown in Fig. 2. When the first derivative of the fitted polynomial is 0, the corresponding *Z*_f_ is the one corresponding to the extreme point of $U^{3} /H$. Then the *D*_f_ corresponding to this *Z*_f_ was queried using the relationship between the *Z*_f_ and *D*_f_ at a nearby hydrographic station. This *D*_f_ is *D*_cf_.

## Results

### ***D***_cf_ in different river sections

Table 2 shows the *D*_cf_ and its changes along the MLYR before and after the TGR impoundment, calculated using the method proposed in this study. As seen in the table, the decreased magnitude of *D*_cf_ from before to after the TGR impoundment was generally between 2500 and 4700 m^3^/s, with the Zhicheng hydrographic station having the largest reduction and the Jianli hydrographic station the smallest. The reduction in the section below Chenglingji was approximately 3000 m^3^/s.

**Table 2:** *D*_cf_ and its changes in the MLYR before and after the impoundment of TGR.

Hydrographic station	*D*_cf_ (m^3^/s)			Representative cross section
	Before TGR	After TGR	Changes	
Zhicheng	34,900	30,200	− 4700	Jing6
Shashi	30,500	27,200	− 3300	Jing28, Jing58
Jianli	27,500	25,000	− 2500	Jing144, Jing177
Luoshan	35,000	31,700	− 3300	CZ04 − 1, JieZ3 + 3, CZ10 − 1
Hankou	43,000	40,200	− 2800	CZ58 − 1, CZ72, CZ76 − 1
Jiujiang	44,000	40,700	− 3300	CZ108 − 1, CZ118
Datong	50,500	47,700	− 2800	CX63 + 69, CX85, CX178

### Examination of ***D***_cf_

**Figure 5:**
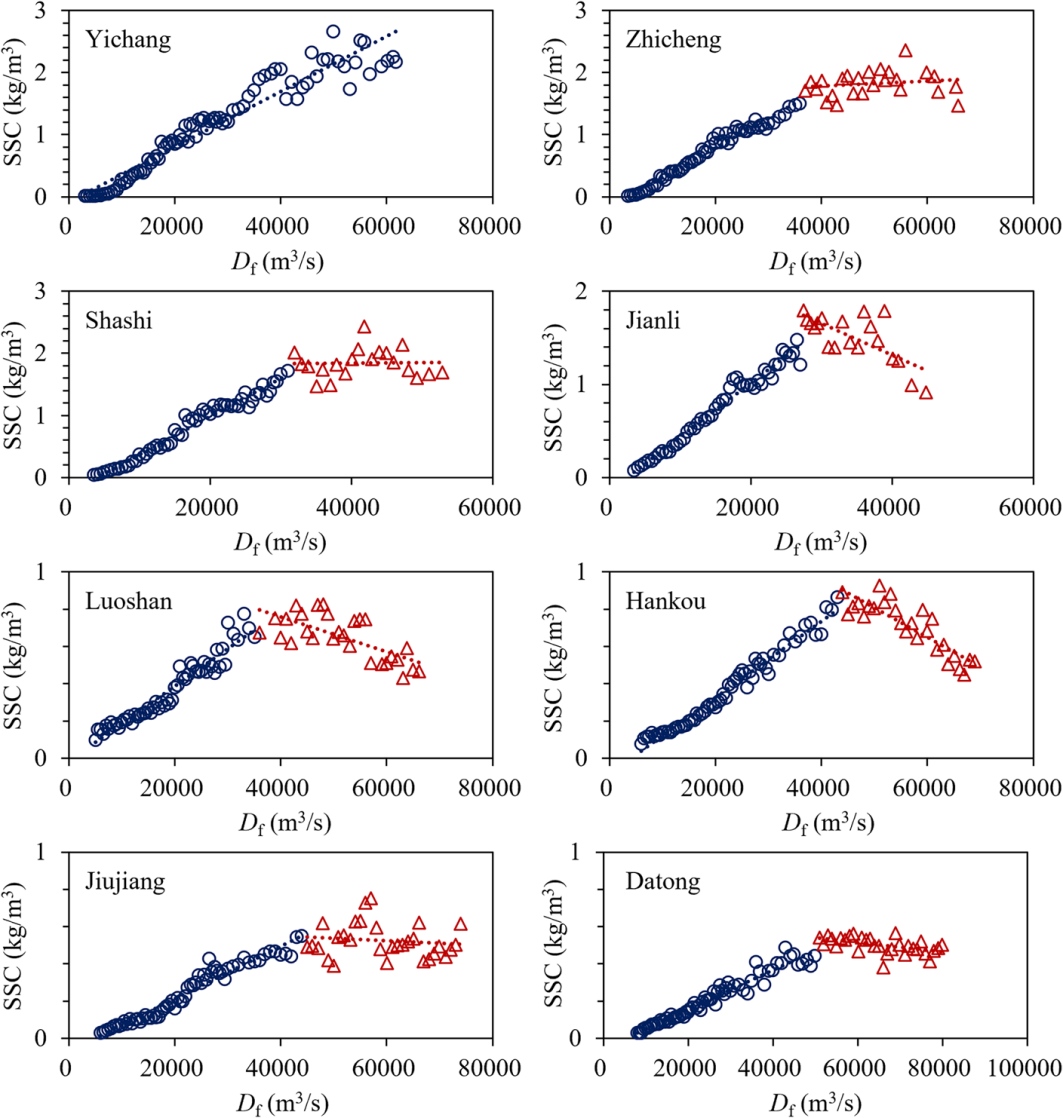
Relationship between *D*_f_ and SSC in MLYR before the TGR impoundment (1981–2002).

**Figure 6:**
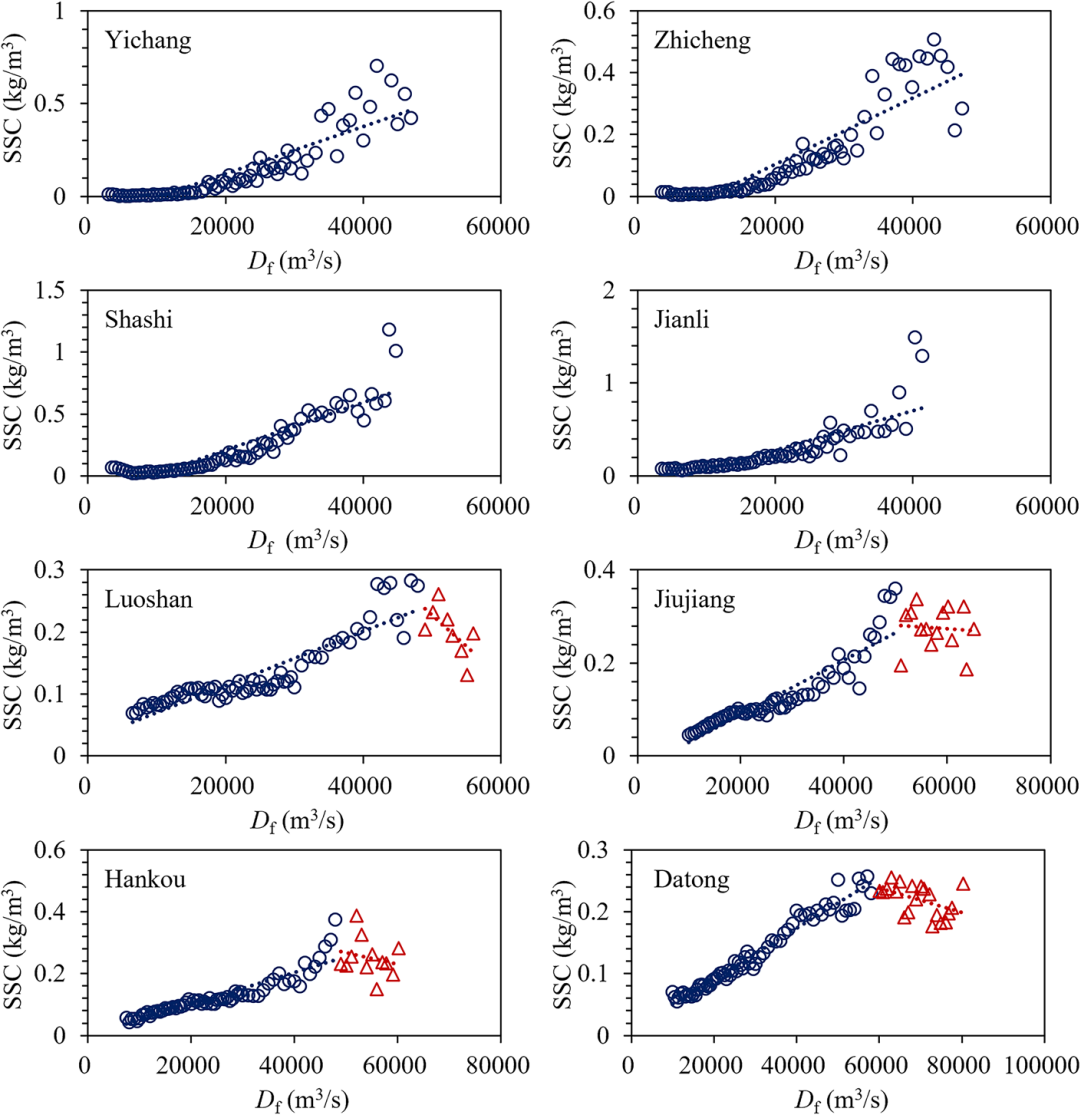
Relationship between *D*_f_ and SSC in MLYR after the TGR impoundment (2003–2020).

The key to the method in this study is to determine the extreme value of the SSCC index, and there are requirements for the shape of the cross sections. This method is not applicable to a single channel without sandbars. To examine the reasonableness of this method, we further established the correlation relationship between *D*_f_ and SSC in the MLYR during 1981–2002 (Fig. 5). During this period, the MLYR was in a state of relative equilibrium between sediment erosion and siltation^52^. The correlations have turning points. The SSC corresponding to this turning point can be considered the SSCC, and the corresponding *D*_f_ has the strongest ability to shape the riverbed. This relationship does not exist or is not evident after the impoundment of the TGR (2003–2020), meaning that the entire MLYR is in a state of subsaturated sediment transport, and the SSC is far from the SSCC. There were no obvious trend turning points in the relationship after the impoundment of the TGR (Fig. 6). Therefore, this method was examined using only the data before the impoundment of the TGR.

Before the impoundment of the TGR, the characteristics of the alluvial plain river began to appear in the river section downstream of Zhicheng. As seen in Fig. 5, the turning features of this relationship are becoming increasingly apparent. The *D*_f_ at the stations corresponding to the turning point was very close to the *D*_cf_ calculated using this method, as shown in Table 2. This indicated that the proposed method was reasonable.

## Discussion

### Comparison of different methods

**Figure 7:**
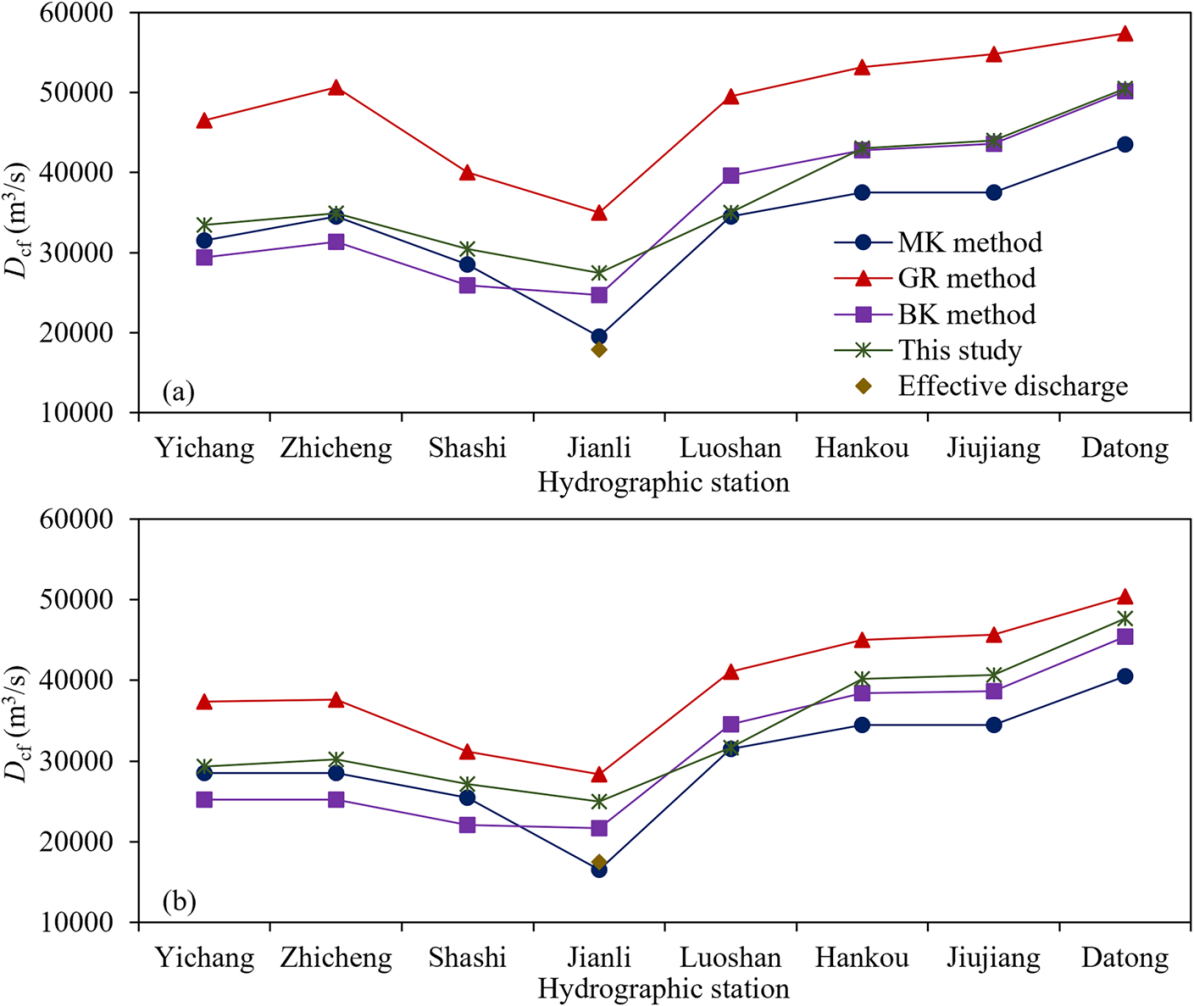
*D*_cf_ calculated by different methods: (**a**) before the TGR; (**b**) after the TGR.

Based on the concept that the evolution of a river channel is subject to water and sediment movement, this study presents a method for calculating *D*_cf_ by identifying the extreme points of the SSCC index. The method is based on the cross-sectional topography and the relationship between the *Z*_f_ and *D*_f_ data of the studied river section, and is relatively simple, but with a good physical background. To test the rationality of this method, Fig. 7 shows a comparison of the *D*_cf_ calculated by the BK, GR, and MK methods, and the method proposed in this study, as well as the effective discharge. Table 3 lists the differences before and after water storage in the TGR.

**Table 3:** Difference in *D*_cf_ before and after the TGR.

Hydrographic station	MK method	GR method	BK method	Effective discharge^12^	This study
Yichang	− 3000	− 9100	− 4200	–	− 4200
Zhicheng	− 6000	− 13,100	− 6200		− 4700
Shashi	− 3000	− 8800	− 3800		− 3300
Jianli	− 3000	− 6600	− 3000	− 400	− 2500
Luoshan	− 3000	− 8400	− 5000	–	− 3300
Hankou	− 3000	− 8200	− 4400		− 2800
Jiujiang	− 3000	− 9100	− 4900		− 3300
Datong	− 3000	− 7000	− 4800		− 2800

Comparison of the results calculated by different methods revealed that before and after storage of the TGR, the *D*_cf_ calculated by the GR method exceeded those calculated by the other three methods. Specifically:

1. The *D*_cf_ calculated using the MK method considers two factors: the SSCC of the *D*_f_ and the duration of different *D*_f_, thus providing a theoretical basis and physical significance. However, the drawback of this method is that the relative change value is often limited by *D*_f_ classification, which makes it difficult for the method to reflect the changes along the river channel. The effective discharge^12^ at Jianli station is relatively close to the MK method result. In addition, it shows that the effective discharge at Yichang station decreases with an amplitude ranging from 15,000 to 20,000 m^3^/s, and 5000 to 10,000 m^3^/s at Shashi station after 2002^13^.
2. The GR method is based on experience in selecting the flood *D*_f_ during a certain recurrence period as *D*_cf_. This method exhibits strong empiricism and subjectivity. However, a water depletion phenomenon has been observed in the MLYR after the construction of the TGR. Although the results obtained by this method can reflect the changing trend along the river channel, they depend solely on the flow process and do not reflect the response of the river boundary conditions.
3. The starting point of the BK method is the change in *Z*_f_, which is often influenced by the water, sediment, and boundary conditions of the reach. This method can compensate for the shortcomings of the MK method by responding to changes along the river reach, but has two limitations. When selecting the terrain of the river channel at a certain period to determine the bankfull elevation at different positions, a large individual error is often made. In addition, most of the sandbars in the MLYR have implemented protection projects, which are influenced by both up-input water and sediment as well as human activities. Thus, it cannot fully reflect the shaping effect of water and sediment, leading to errors in the results.
4. The method proposed in this study considers both the shaping effect of water flow and the response of the riverbed, and is simultaneously verified using the characteristics of water flow loaded by sediment. The results are between those of the MK and BK methods and reflect the characteristics of changes along the river channel. The limitation of this method is that the studied river sections should have clearly distinguishable sandbars and deep pools. In this type of river, the synergistic effect of water flow and sediment in shaping the riverbed is relatively pronounced^53^, which makes it easier to detect *D*_cf_.

### Influencing factors of ***D***_cf_

The water situation in the Yangtze River Basin has a certain periodicity. After the TGR impoundment, the upper reaches of the Yangtze River enter a hydrological cycle of runoff depletion. The insufficient precipitation leads to a corresponding decrease in the runoff entering the MLYR, which in turn leads to a reduction in the source of sediment in the river. Therefore, the relatively dry hydrological cycle of the main stream of the Yangtze River after the TGR impoundment is one of the important factors for the smaller calculated values of *D*_cf_ in the MLYR compared to before the impoundment.

The sediment interception effect of reservoir operation has led to a reduction of over 98% in sediment transport at Yichang station in recent years, which will have a direct impact on the parameter values in the MK method. With the gradual implementation of dry season replenishment, peak shaving during flood season, and early storage after flood season in reservoirs, the impact of reservoirs on the downstream river runoff process is becoming increasingly apparent. The replenishment during dry season and early storage after flood season mainly affect the flow of small and medium water, and have a relatively small impact on *D*_cf_. The peak shaving scheduling during the flood season changes the natural flood process, with the most obvious being the reduction of peak flow. Therefore, when using the MK or GR methods to calculate *D*_cf_, the values after the TGR should be slightly smaller.

Reservoir regulation not only directly changes the water and sediment conditions, but also brings about changes in the river boundary. The riverbed is generally eroded, and the cross-sectional shape, longitudinal profile shape, sandbar shape, and even bed surface shape will be adjusted accordingly. The ultimate goal of these adjustments is to reduce the flow velocity, match the SSCC with the incoming SSC, and thus make the river tend towards an equilibrium state. In this sense, after the TGR impoundment, the *D*_cf_ downstream the dam should also decrease, thereby promoting the evolution of the river towards a balanced state.

To stabilize the river channel conditions, remediation projects, most of which are in the form of protection, have been implemented. In addition, embankments have been built to develop and utilize the sandbars. These projects have altered the mobility of local river boundaries and disrupted the natural and continuous response of protected geomorphic units to water and sediment processes. When determining the *D*_cf_ using the BK method or that proposed in this study, the deformation of the sandbars is limited and often cannot reflect the response relationship between sandbar erosion and changes in water and sediment conditions.

### Implications on reservoir and river management

The operation of TGR, controlling reservoir in the upper reaches of the Yangtze River needs to ensure the flood safety of the reservoir and the downstream river channel, reduce sedimentation in the reservoir area, and ensure the development of the downstream river channel of the dam. This indicates that when encountering floods, it is not only necessary to control the peak flood *D*_f_ and total flood volume in combination with the reservoir flood control capacity, but also to maintain a certain *D*_f_ during the flood season to increase the sediment output capacity of the reservoir. In addition, the output *D*_f_ needs to reach the *D*_cf_ and maintain it for a certain period of time.

Since the TGR entered the experimental storage stage, peak shaving operations have been carried out for flood peaks exceeding 45,000 m^3^/s. The frequency of high floods occurring downstream of the dam has decreased, and the overflowing probability for high sandbars has decreased correspondingly. Comparison of the calculation results of different methods reveals that compared to before the water storage of TGR, the *D*_cf_ downstream the dam has decreased after the water storage, and the magnitude of reduction is smaller as it moves downstream. The actual evolution shows that the adjustments to the cross-sectional shape, longitudinal profile shape, and sandbars shape downstream the dam are within the expected range of changes. Certain branching channels have also exhibited expected shrinkage, and the reduction in *D*_cf_ is acceptable.

In the long run, maintaining the current peak shaving operation of the reservoir can achieve various goals such as flood control and reservoir sedimentation. However, at the same time, the output *D*_f_ exceeding the* D*_cf_ downstream of the dam with a certain duration within the year should be satisfactory. In each hydrological cycle, there should be a certain number of years that meets the duration of *D*_cf_ to avoid further reduction and ensure the normal development of the river. For the river management downstream reservoirs, full consideration should be given to the actual situation of reduced* D*_cf_ to develop effective response measures. This study provide a new method for determining the *D*_cf_. However, the specific duration of *D*_cf_ and its total number of years in a hydrological cycle need to be analyzed in greater detail in future research studies.

## Conclusions

By combining the actual river evolution in the MLYR and considering the water, sediment and riverbed boundary, this study proposed a method for determining *D*_cf_ by identifying extreme value of the SSCC index. The results indicated that this extreme value is easily detected in the MLYR. Calculations show that after the impoundment of TGR, the *D*_cf_ along the MLYR decreases to varying degrees, with an average of approximately 3360 m^3^/s. The maximum reduction (4700 m^3^/s) occurred in Zhicheng, followed by Yichang with 4200 m^3^/s. The reduction in Shashi, Luoshan, and Jiujiang stations were 3300 m^3^/s, while those in Hankou and Datong stations were 2800 m^3^/s. Jianli station reported the lowest reduction: 2500 m^3^/s.

In the river downstream of the reservoir, water and sediment conditions, and river boundaries can be derived by specific hydrological cycle changes, reservoir scheduling and operation, river management, and the development and utilization of sandbars. Among these, the focus was on hydrological cycle changes and reservoir operations. The former mainly affects the conditions of water and sediment, whereas the latter also affects the changes the river boundary conditions. The implementation of large-scale river regulation projects also has a significant impact on *D*_cf_. The method proposed in this study is expected to provide *D*_cf_ in downstream river regulation and reservoir operation to better leverage the comprehensive benefits of the reservoir and improve the effectiveness of downstream river regulation projects.

## Data Availability

The datasets used in this study are available from the corresponding author upon request.

## References

[1] Fola ME, Rennie CD (2010). Downstream hydraulic geometry of clay-dominated cohesive bed Rivers. J. Hydraul. Eng..

[2] Wang X, Wang D, Zhao L (2008). Discussion on the applicability of bed-forming discharge method in calculating regulation water level. Port Waterway Eng..

[3] Gorney RM, Ferris DR, Ward AD, Williams LR (2011). Assessing channel-forming characteristics of an impacted headwater stream in Ohio, USA. Ecol. Eng..

[4] Wolman MG, Miller JP (1960). Magnitude and frequency of forces in geomorphic processes. J. Geol..

[5] Lawlor, S. M. Determination of channel-morphology characteristics, bankfull discharge, and various design-peak discharges in western Montana. Report No. 2004-5263 (2004).

[6] Surian N, Mao L, Giacomin M, Ziliani L (2009). Morphological effects of different channel-forming discharges in a gravel-bed river. Earth Surf. Process. Landf..

[7] Schuurman F (2018). Response of braiding channel morphodynamics to peak discharge changes in the Upper Yellow River. Earth Surf. Process. Landf..

[8] Powell GE, Mecklenburg D, Ward A (2006). Evaluating channel-forming discharges: A study of large rivers in Ohio. Trans. Asabe.

[9] Ward A, Moran M (2016). A novel approach for estimating the recurrence intervals of channel-forming discharges. Water.

[10] Pickup G, Warner RF (1976). Effects of hydrologic regime on magnitude and frequency of dominant discharge. J. Hydrol..

[11] Bao W, Guo W, Li X, Qu T (2018). Study on dominant discharge, bankfull discharge and effective discharge of Liaohe River. J. Sediment Res..

[12] Chen D, Yu M, Zhu Y (2018). Study on effective discharge in the Lower Jingjiang River before and after construction of the Three Gorges Dam. Adv. Water Sci..

[13] Yang X, Xiong H, Li D, Li Y, Hu Y (2023). Disproportional erosion of the middle-lower Yangtze River following the operation of the Three Gorges Dam. Sci. Total Environ..

[14] H.N., M. & Zhou, Z. An approximate method for determining the bed making flow rate. *Yangtze River*, 52–53, 10.16232/j.cnki.1001-4179.1957.11.016 (1957).

[15] H.N., M. & Mai, Q. Channel forming discharge. *J. Sediment Res.* 40–43, 10.16239/j.cnki.0468-155x.1957.02.005 (1957).

[16] Shen M, Shi H, Guo Q, Lu Q (2021). Study on characteristics of sediment transport and channel-forming discharge above the Karun Mountain in the Heilongjiang River. J. Sediment Res..

[17] Yu B, Yu Y, Zhao K (2010). Calculation of dominant discharge in the middle reaches of Huaihe River. J. Hohai Univ. (Nat. Sci.).

[18] Feng H (2011). A study on the method for determining bedding flow in the near dam runoff regulation river section. China Water Transp..

[19] Chen J, Hu C, Dong Z, Liu D (2006). Change of bankfull and bed-forming discharges in the lower Yellow River. J. Sediment Res..

[20] Chu W, Li Y, Xie L (2015). Calculation of dominant discharge of Chongqing urban reach in Yangtze river main stream. J. Chongqing Jiaotong Univ. (Nat. Sci.).

[21] Duan H, Lu Q, Wang Z, Yang D, Li X (2023). Analysis of calculation method of bed-forming flow in the lower reaches of the Yellow river. Water Resour. Power.

[22] Annable WK, Lounder VG, Watson CC (2011). Estimating channel-forming discharge in urban watercourses. River Res. Appl..

[23] Doyle MW, Shields D, Boyd KF, Skidmore PB, Dominick D (2007). Channel-forming discharge selection in river restoration design. J. Hydraul. Eng.-Asce.

[24] Zhang W, Xu J (2017). Study on dominant discharges in the lower tidal reach of the Yangtze River. Port Waterway Eng..

[25] Wu Y, Jia Y, Fan B (2008). Analysis of dominant discharge on the middle and lower reaches of the Songhua River. J. Harbin Inst. Technol..

[26] Wang J, Qin G, Wang X, Tong X (2019). Discussion on bed-building discharge calculation in mountainous river. Yangtze River.

[27] Ji Z, Hu C, Yan Y, Niu J (1994). Dominant discharge on heavy sediment-laden river. Adv. Water Sci..

[28] Han Q (2009). Theoretical analysis on first bed-forming discharge and sediment transporting capacity. Yellow River.

[29] Han Q, Jiang E, Chen X (2009). Study on second bed-forming discharge of the lower Yellow river. Yellow River.

[30] Chen X, Han Q, Fang C (2007). Variation of dominant discharge in lower Yellow River and its influence on river channel. J. Hydraul. Eng..

[31] Zhang H, Zhang Q, Jiang E (1994). Calculation of dominated discharge in the lower Yellow river. J. Sediment Res..

[32] Dai S, Han J, Cao Q, Li C (2020). Spatial and temporal distribution of dominant discharge and effective discharge in the lower reaches of Weihe river. Res. Soil Water Conserv..

[33] Blom A, Arkesteijn L, Chavarrías V, Viparelli E (2017). The equilibrium alluvial river under variable flow and its channel-forming discharge. J. Geophys. Res.-Earth Surf..

[34] Lai Z (2020). Study on dominant discharge change in the lower Yellow River based on moving analysis method. J. Centr. South Univ. (Sci. Technol.).

[35] Sun D, Wang Q, Wang P, Zhang X (2013). Determination of dominant discharge of the lower Yellow River based on coefficient frequencies of flow-sediment relationship. J. Hydroelectr. Eng..

[36] Li F, Gao L (1990). Preliminary study on the calculation method of bedmaking flow rate. J. Hohai Univ. (Nat. Sci.).

[37] Xu J (2022). Variation of dominant discharge along the riverbed based on numerical and deep-learning models: A case study in the Middle Huaihe River, China. J. Hydrol..

[38] Zhang C, Yi X, Hu T, Zhang C (2009). Analysis on formative discharge and cross-sectional adjustment of the Sanmenxia reservoir. Yellow River.

[39] Chen L, Lu Q, Wang Z (2023). Processes of bed-forming flow and its driving factors at Toudaoguai Hydrological Station in the main stream of the Yellow River. J. Sediment Res..

[40] Wang K, Liu X, Zhang H, Zhang X (2023). Variation of bed-forming discharge and its influencing factors in the inner Mongolia reach of the Yellow river. J. Yangtze River Sci. Res. Inst..

[41] Sun Z (2021). Relationship between the characteristics of water-sediment transportation in river-lake system and the channel forming discharge of the middle and lower Yangtze River. J. Hydraul. Eng..

[42] Zhou W, Sun Z, Zhou K, Li Z, Chen L (2022). Characteristics of discharge frequency and their effects on flood channel formation in the middle and lower reaches of the Yangtze River before/after the impoundment of the Three Gorges Reservoir. J. Lake Sci..

[43] Sholtes J, Werbylo K, Bledsoe B (2014). Physical context for theoretical approaches to sediment transport magnitude-frequency analysis in alluvial channels. Water Resour. Res..

[44] Yan J, Tang Q, Zou T (2014). Variation of dominant discharge and sediment-carrying capacity of flow in the downstream of three gorges reservoir. J. Yangtze River Sci. Res. Inst..

[45] Yang Y, Zhou L, Zhu L, Liu W, Wang J (2023). Impact of upstream reservoirs on geomorphic evolution in the middle and lower reaches of the Yangtze River. Earth Surf. Process. Landf..

[46] Yang Y, Zheng J, Zhu L, Zhang H, Wang J (2022). Influence of the Three Gorges Dam on the transport and sorting of coarse and fine sediments downstream of the dam. J. Hydrol..

[47] Li Z (2021). Channel morphologic processes of a highly sinuous bend approaching neck cutoff by bank erosion in the middle Yangtze River. Int. J. Sediment Res..

[48] Zhu B, Li Y, Yue Y, Yang Y (2017). Aggravation of north channels' shrinkage and south channels' development in the Yangtze Estuary under dam-induced runoff discharge flattening. Estuarine, Coast. Shelf Sci..

[49] Zhu B (2020). Alternate erosion and deposition in the Yangtze Estuary and the future change. J. Geogr. Sci..

[50] Li J (2023). Sediment deposition within cascade reservoirs: A case study of Baihetan Reservoir in the lower Jinshajiang River, China. Sci. Rep..

[51] Ge, H., Deng, C. & Gong, P. in *2021 4th International Conference on Civil, Architecture and Environment Research, ICCAER 2021, January 15, 2021 - January 17, 2021.*1 edn (IOP Publishing Ltd).

[52] Xu Q, Zhu L, Yuan J (2013). Research on water-sediment variation and deposition-erosion in middle and lower Yangtze River. Yangtze River.

[53] Hu P (2021). Role of bar-channel interactions in a dominant branch shift: The Taipingkou waterway, Yangtze River, China. River Res. Appl..

